# The effectiveness and safety of Simiao Xiaobi decoction on rheumatoid arthritis: A study protocol for systematic review and meta-analysis

**DOI:** 10.1097/MD.0000000000029174

**Published:** 2022-03-25

**Authors:** Soo-Yeon Chae, Yeonju Woo, Joo-Hee Kim, Eun-Jung Kim, Byung-Kwan Seo, Yong-Hyeon Baek, Seong-Sik Park, Won-Suk Sung

**Affiliations:** a *College of Korean Medicine, Dongguk University Graduate School, Seoul, Republic of Korea,*; b *Department of Physiology, College of Korean Medicine, Sangji University, Wonju-si, Gangwon-do, Republic of Korea,*; c *Department of Acupuncture and Moxibustion Medicine, College of Korean Medicine, Sangji University, Wonjusi, Gangwon-do, Republic of Korea,*; d *Department of Acupuncture & Moxibustion, Dongguk University Bundang Oriental Hospital, Seongnam-si, Gyeonggi-do, Republic of Korea,*; e *Department of Acupuncture & Moxibustion, Kyung Hee University Hospital at Gangdong, Seoul, Republic of Korea,*; f Department of Sasang Constitutional Medicine, Dongguk University Bundang Oriental Hospital, Seongnam-si, Gyeonggi-do, Republic of Korea.

**Keywords:** meta-analysis, randomized controlled trials, rheumatoid arthritis, Simiao Xiaobi decoction, systematic review

## Abstract

**Background:**

Rheumatoid arthritis (RA) is a common chronic autoimmune disease that contributes to progressive disability, systemic complications, higher mortality, and societal burden. Typical symptoms of RA include symmetrical pain and swelling in multiple joints, morning stiffness, and elevated levels of erythrocyte sedimentation rate, C-reactive protein, and rheumatoid factor. The representative treatment for RA is medication, including disease-modifying antirheumatic drugs, glucocorticoids, and nonsteroidal anti-inflammatory drugs. However, these medications are not yet curative nor preventative and are associated with several adverse effects, leading to their discontinuation. Recent articles reported that Simiao Xiaobi decoction (SXD) could relieve the symptoms of RA by clinical trial and experimental study, but an evidence-based review on the effectiveness and safety of SXD on RA has not yet been provided.

**Methods:**

Searching for randomized controlled trials on the use of SXD for RA will be performed by using multiple electronic databases, manual search, and contacting the authors by e-mail if needed. Studies will be selected according to the predefined criteria and the data collected on study participants, interventions, control groups, outcome measurements, their results, adverse events, and risk of bias will be summarized. The primary outcome will be the disease activity score (including effective rate, swollen joint count, tender joint count, and morning stiffness), and the secondary outcomes will be blood tests (including erythrocyte sedimentation rate, C-reactive protein, and rheumatoid factor) and adverse events. We will use Review Manager software to perform a meta-analysis, the Cochrane Collaboration “risk of bias” tool for assessing the risk of bias, and grades of recommendation, assessment, development and evaluation for the determination of the quality of evidence.

**Trial registration number:**

https://inplasy.com; INPLASY202230026.

**Results:**

We are going to investigate the effectiveness and safety of SXD for RA.

**Conclusion:**

This study will provide reliable evidence on whether SXD is effective on RA.

## 1. Introduction

Rheumatoid arthritis (RA) is a common chronic autoimmune disease with a global prevalence estimate of 0.46%,^[1]^ that contributes to progressive disability, systemic complications, higher mortality, and societal burden.^[2-4]^ The etiology is still unclear, but it is believed that multiple genetic and environmental factors lead to hyper-responsive condition of immune cells against self-antigens, leading to inflammation, and joint damage.^[5]^ Typical presentations of RA include symmetrical pain and swelling in multiple joints, accompanied by morning stiffness lasting more than 30 minutes. Elevated levels of rheumatoid factor and acute phase reactant response, including increased C-reactive protein and erythrocyte sedimentation rate, are also included in the diagnosis criteria.^[6]^

Current treatments for RA have the purpose of treating symptoms by alleviating pain, reducing inflammation, and delaying disease progression through suppressing immune players and inflammatory mediators. The representative treatment for RA is medication, including glucocorticoids (GCs), nonsteroidal anti-inflammatory drugs (NSAIDs), and diseasemodifying antirheumatic drugs (DMARDs).^[7,8]^ However, these medications are not yet curative or preventative.^[9]^ Some studies have reported that conventional medication induced several adverse effects, leading to discontinuation.^[9]^ The adverse effects of methotrexate, the most commonly used treatment for RA, range from the most common gastrointestinal disorders, which affect 20% to 70% of patients, to severe problems such as hepatitis, pulmonary damage, and myosuppression.^[9]^ Moreover, it is also ineffective in a proportion of patients. Therefore, natural products and other complementary and alternative medicine approaches are gaining interest.^[10]^

Simiao Xiaobi decoction (SXD), composed of multiple herbs, including Jinyinhua (*Lonicera japonica*), Danggui (*Radix Angelicae Sinensis*), Baihuasheshecao (*Hedyotis diffusa*), and Baishao (*Radix Paeoniae Alba*), traditionally used for the treatment of RA. In clinical trial, SXD showed better effective rate and superior improvements in RA-related symptoms and sign scores than the control group receiving methotrexate.^[11]^ Regarding experimental studies, Jinyinhua was reported to show therapeutic effects against RA by exerting potent chondroprotective and anti-inflammatory effects.^[12]^ Baihuasheshecao has been known to include iridoids, flavonoids, and phenolic acids and plays a role in immune response regulation and anti-inflammatory activity.^[13]^ Total glucosides of paeony from the roots of *Paeonia lactiflora Pallas* are known to alleviate unanticipated hepatic adverse effects during conventional treatment of RA.^[14]^

Although there are several studies reporting the effectiveness of SXD on RA, an evidence-based review on the effectiveness and safety of SXD has not yet been provided. In this study, we are going to investigate the effectiveness and safety of SXD for RA.

## 2. Methods

### 2.1. Study design

A systemic review will be conducted according to the preferred reporting items for systemic reviews and meta-analyses protocols 2015 statement.^[15]^

### 2.2. Ethics

Since there will be no requirements for the collection of patients’ or personal information, an ethical statement is not required.

### 2.3. Study registration

The protocol was registered with https://inplasy.com (Registration number: 202230026).

### 2.4. Eligibility criteria

#### 2.4.1. Participants.

Patients who were diagnosed with RA, regardless of age and gender, will be included. Patients with osteoarthritis will be excluded.

#### 2.4.2. Types of interventions.

A randomized controlled trial (RCT) investigating the effects of SXD on RA with various conventional treatments including physiotherapy, occupational therapy, and medication (DMARDs, GCs, and NSAIDs) will be included. The use of combination treatment with SXD in the experimental group should be consistent with control group.

#### 2.4.3. Type of studies.

This study will include RCTs that compare the effects of SXD on RA with a control group containing conventional treatments. RCTs that did not provide the randomization method or conducted randomization incorrectly and uncontrolled clinical trials (eg, observational studies, pilot studies, case studies, and systematic review) will be excluded.

#### 2.4.4. Outcome measures.

The primary outcome will be the disease activity score, including effective rate, swollen joint count, tender joint count, and morning stiffness. Blood tests for RA, including erythrocyte sedimentation rate, C-reactive protein, and rheumatoid factor, and adverse events will be considered as secondary outcomes.

#### 2.4.5. Language.

There will be no limitations according to language.

### 2.5. Information sources and search strategy

MEDLINE, Cochrane Library, China National Knowledge Infrastructure, CiNii, J-STAGE, KoreaMed, Korean Medical Database, Korean Studies Information Service System, National Digital Science Library, Korea Institute of Science and Technology Information, and Oriental Medicine Advanced Searching Integrated System will be used to search for related studies published from the initial time of each database to June 2022. A combination of the following terms will be used to conduct a search, in each database's language (arthritis, rheumatoid or rheumatoid arthritis) and (Simiao Xiaobi decoction or Simaio Xiaobi Tang) and comparison (name of DMARDs or GCs or NSAIDs). More searches of relevant gray literature sources, reports, and dissertations will be conducted. Manual searches like textbooks on SXD and its references and contacting authors via e-mail will be done if needed.

Table 1 shows the search strategy for the MEDLINE via PubMed.

**Table 1:** Search strategy for the MEDLINE via PubMed.

**No.**	**Search terms**
#1	Rheumatoid arthritis
#2	Rheumatoid or arthritis
#3	#1 and #2
#4	Randomized controlled trial or random^*^ or placebo
#5	Controlled clinical trial or trial
#6	Simiao Xiaobi decoction
#7	Simiao Xiaobi tang
#8	#6 or #7
#9	#3 and (#4 or #5) and #8

### 2.6. Study selection

**Figure 1.**
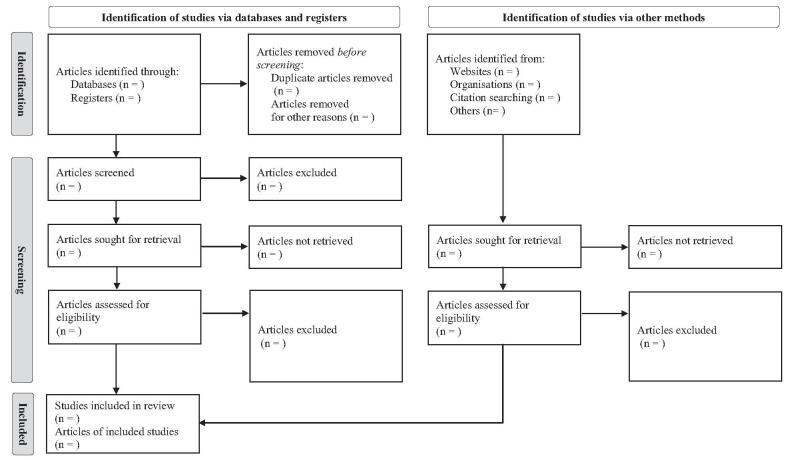
PRISMA flow diagram. PRISMA = preferred reporting items for systemic reviews and meta-analyses.

After the search is conducted, 2 researchers will independently screen through the records for inclusion. Duplicate studies will be excluded first, and more studies will be excluded according to the assessment of title, abstract, and full text. Then, using predefined criteria, 2 reviewers will read the full texts of the potentially eligible articles for the assessment of inclusion, with any conflicts resolved through discussion. If the 2 reviewers fail to reach an agreement, the third reviewer will make the final decision (Fig. 1).

### 2.7. Data management

Management software EndNote X20 (Clarivate Analytics, Philadelphia, PA, USA) will be used for the management of compiled data.

### 2.8. Data extraction

Two reviewers will take part in the extraction of the data in accordance with the predefined criteria. Data on study information, including first author and publication year, patient characteristics, interventions and comparators, outcome measures, the results, and information for the assessment of study quality, will be extracted. Any disagreement on data extraction will be resolved by discussion until consensus is reached or by consulting a third reviewer.

### 2.9. Data synthesis and analysis

We will combine the changes from baseline to completion of the intervention by using the Review Manager (The Cochrane Collaboration, Oxford, UK) software for Windows to perform a meta-analysis (The Cochrane Collaboration, Oxford, UK). We will calculate the mean difference and 95% confidence intervals in the same outcome measures and the standardized mean difference and 95% confidence intervals in different outcome measures to estimate the effect with a random-effect or a fixedeffect model. The heterogeneity assessment will be calculated by Chi-squared and I-squared and will be interpreted as follows; unimportant heterogeneity, 0% to 40%; moderate heterogeneity, 30% to 60%; substantial heterogeneity, 50% to 90%; and considerable heterogeneity, 75% to 100%. If possible, the subgroup analysis will be conducted based on the main intervention in the control group. A narrative synthesis will be conducted if quantitative synthesis is not possible, using the available data. A funnel plot will be used regarding publication bias when there are more than 10 identified studies in the metaanalysis. In rating the quality of evidence for each outcome, the grading of recommendations assessment, development, and evaluation method will be used.^[16]^

### 2.10. Risk of bias assessment

The risk of bias will be assessed by 2 reviewers independently using the Cochrane “risk of bias” tool.^[17]^ The tool is composed of 7 domains: sequence generation, allocation concealment, blinding of participants and investigators, blinding of outcome assessors, incomplete outcome data, selective outcome reporting, and other biases. The risk of bias will be rated as “low risk”, “high risk”, or “unclear risk” for each domain.

## 3. Discussion

Conventional treatments for RA have been helpful, but there are limitations and adverse effects to consider. Thus, an effort to find a safer, more effective method of treating RA is in the process. SXD has been clinically verified by previous research and has the potential to show good performance. This study will provide an evidence-based review for patients, clinicians, policy makers, and researchers.

## Author contributions

**Conceptualization:** Eun-Jung Kim, Won-Suk Sung.

**Funding acquisition:** Joo-Hee Kim.

**Investigation:** Soo-Yeon Chae.

**Methodology:** Eun-Jung Kim, Byung-Kwan Seo, Yong-Hyeon Baek, Seong-Sik Park.

**Project administration:** Joo-Hee Kim, Byung-Kwan Seo.

**Supervision:** Won-Suk Sung.

**Writing** - **original draft:** Soo-Yeon Chae.

**Writing** - **review & editing:** Yeonju Woo, Yong-Hyeon Baek, Seong-Sik Park, Won-Suk Sung.
